# Treatment of Multidrug-resistant or Rifampicin-resistant Tuberculosis With an All-oral 9-month Regimen Containing Linezolid or Ethionamide in South Africa: A Retrospective Cohort Study

**DOI:** 10.1093/cid/ciae145

**Published:** 2024-03-25

**Authors:** Hannah Morgan, Norbert Ndjeka, Tasnim Hasan, Medea Gegia, Fuad Mirzayev, Linh N Nguyen, Samuel Schumacher, Timothy E Schlub, Kogieleum Naidoo, Greg J Fox

**Affiliations:** Faculty of Medicine and Health, University of Sydney, Sydney, NSW, Australia; National Department of Health, Tuberculosis Control and Management Cluster, Pretoria, South Africa; Faculty of Medicine and Health, University of Sydney, Sydney, NSW, Australia; Global Tuberculosis Program, World Health Organisation, Geneva, Switzerland; Global Tuberculosis Program, World Health Organisation, Geneva, Switzerland; Global Tuberculosis Program, World Health Organisation, Geneva, Switzerland; Global Tuberculosis Program, World Health Organisation, Geneva, Switzerland; Faculty of Medicine and Health, University of Sydney, Sydney, NSW, Australia; Nelson R Mandela School of Medicine, University of KwaZulu Natal, Durban, South Africa; Faculty of Medicine and Health, University of Sydney, Sydney, NSW, Australia

**Keywords:** multidrug- resistant tuberculosis, ethionamide, linezolid, all-oral 9-month regimen

## Abstract

**Background:**

In 2019, the South African tuberculosis program replaced ethionamide with linezolid as part of an all-oral 9-month regimen. We evaluated treatment outcomes for patients assigned to regimens including linezolid in 2019 and ethionamide in 2017.

**Methods:**

This retrospective cohort study included patients treated for multidrug-resistant/rifampicin-resistant tuberculosis throughout South Africa between 1 January and 31 December 2017 and 1 January to 31 December 2019. The cohort treated with a 9-month regimen containing ethionamide for four months, was compared with a cohort treated with a 9-month regimen containing linezolid for 2 months. The regimens were otherwise identical. Inverse probability weighting of propensity scores was used to adjust for potential confounding. A log-binomial regression model was used to estimate adjusted relative risk (aRR) comparing 24-month outcomes between cohorts including treatment success, death, loss to follow up, and treatment failure. Adverse event data were available for the linezolid cohort.

**Findings:**

In total, 817 patients were included in the cohort receiving ethionamide and 4244 in the cohort receiving linezolid. No evidence for a difference was observed between linezolid and ethionamide regimens for treatment success (aRR = 0.96, 95% confidence interval [CI] .91–1.01), death (aRR = 1.01, 95% CI .87–1.17) or treatment failure (aRR = 0.87, 95% CI .44–1.75). Loss to follow-up was more common in the linezolid group, although estimates were imprecise (aRR = 1.22, 95% CI .99–1.50).

**Conclusions:**

No significant differences in treatment success and survival were observed with substitution of linezolid for ethionamide as a part of an all-oral 9-month regimen. Linezolid is an acceptable alternative to ethionamide in this shorter regimen for treatment of multidrug-resistant/rifampicin-resistant tuberculosis.

## Teaser

The effectiveness of a 9-month regimen containing linezolid for treating multidrug-resistant tuberculosis was evaluated against the previously recommended regimen containing ethionamide. In a cohort of 5061 patients, no significant differences in treatment success and survival were observed between regimens.

Global treatment guidelines for multidrug-resistant (MDR)/rifampicin-resistant (RR) tuberculosis (TB) have changed considerably over the past 5 years. Novel and repurposed antibiotics, including bedaquiline and linezolid, have replaced injectable agents [1, 2]. Standard treatment has been reduced from 18 to 20 months to between 6 and 12 months for selected patients. In 2019, the World Health Organisation (WHO) endorsed a 9-month all-oral regimen containing bedaquiline for the treatment of MDR/RR-TB among patients with fluoroquinolone-susceptible disease [1]. Recommendations were based on observational data from South Africa indicating improved effectiveness and improved tolerability for shorter-course regimens [3].

In South Africa, the National TB program has implemented 2 novel bedaquiline-containing regimens for treatment of MDR/RR-TB since 2017 [4]. These shorter regimens are 9-months duration, with the option to extend to 11 months based on treatment response. The first regimen includes seven drugs (bedaquiline, clofazimine, a fluoroquinolone, ethionamide, high-dose isoniazid, pyrazinamide, and ethambutol) [5]. Concerns regarding efficacy and poor tolerability of ethionamide [5, 6] and a study of drug resistance patterns in South Africa indicating resistance to ethionamide in up to 45% of patients with MDR/RR-TB [7], led to a decision of linezolid replacing ethionamide within this standard regimen.

Linezolid is a repurposed oxazolidinone antibiotic with early bactericidal activity against *M. tuberculosis* [8, 9]. It is associated with improved treatment outcomes and a reduction in mortality [6, 10]; however, it frequently causes toxicity (myelosuppression, peripheral neuropathy, and optic nerve damage), and higher doses are often poorly tolerated [11].

We undertook a retrospective cohort study to compare 24-month outcomes between patients receiving a 9-month all-oral regimen including either ethionamide or linezolid within the South African National TB Program. A secondary aim was to assess tolerability of a linezolid-containing regimen in a programmatic context. These analyses informed 2022 updates to the WHO MDR/RR-TB treatment guidelines.

## METHODS

### Study Population

Treatment of MDR/RR-TB in South Africa is decentralized, and data collected across 9 provinces [12]. The all-oral 9-month regimen is implemented programmatically with centralised oversight, and data were reported in a national database (EDRweb) [4, 5, 13].

### Eligibility Criteria for 9-month Regimen

Eligibility for the 2 regimens was defined in national guidelines [5]. To be eligible for a shorter all-oral course, individuals required a diagnosis of pulmonary TB or uncomplicated extrapulmonary TB that (i) was caused by *M. tuberculosis,* (ii) with resistance to rifampicin, regardless of isoniazid resistance, (iii) with no more than 1-month prior exposure to second-line treatment for RR-TB, and (iv) with no evidence of resistance to a fluoroquinolone or an injectable antibiotic (for additional details see Supplementary Appendix p2).

Rifampicin resistance was initially identified using GeneXpert MTB/RIF and confirmed on Line Probe Assay testing (LPA). Mutations conferring isoniazid resistance, including *inhA* and *katG* mutations, were identified using first-line LPA. The presence of both mutations was an exclusion criterion. Susceptibility to fluroquinolones and injectable agents was tested using second-line LPA. Where susceptibility was found, phenotypic testing was carried out for fluroquinolone resistance. From 2019, additional testing for resistance to bedaquiline and clofazimine was performed [5].

### Eligibility for Inclusion into the Study Cohort

Study patients were included in the analysis if: (i) treatment duration did not exceed 365 days, (ii) bedaquiline was included in the regimen, and (iii) ethionamide was included (for the 2017 regimen) or linezolid included (for the 2019 regimen). Exclusion criteria included: (i) receipt of <5 drugs, in keeping with contemporaneous WHO guidelines [14]; (ii) a cure or completion outcome was reported prior to 250 days (ie < 8 months), or (iii) an injectable antibiotic was used during treatment.

### Description of Regimens

The all-oral 9-month regimen used in South Africa in 2017 involved bedaquiline, levofloxacin/moxifloxacin, clofazimine, ethionamide, ethambutol, isoniazid (high dose), and pyrazinamide [5] (for regimen description see Supplementary Appendix p2).

The all-oral 9-month regimen used in 2019 was identical to the ethionamide-containing regimen, except linezolid (600 mg daily) was given at the start of treatment and stopped after 2 months, in place of ethionamide. All other drugs and dosing remained the same [5].

### Data Extraction

**Figure 1.**
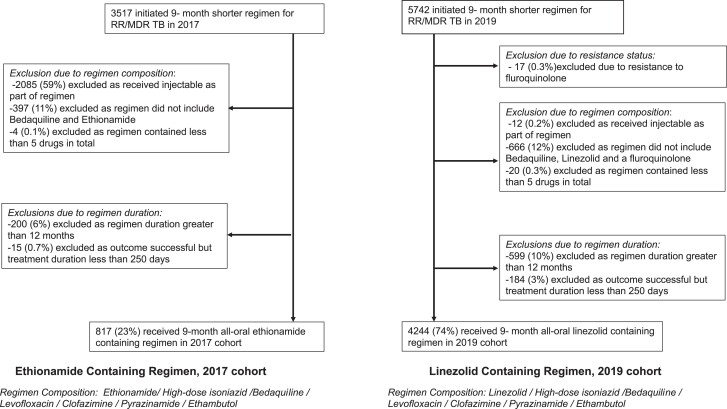
Flow diagram of selection of study participants from patients notified with RR-TB allocated to a 9-month regimen within the South Africa national TB program. Abbreviations: MDR-TB, multidrug-resistant tuberculosis; RR-TB, rifampicin-resistant tuberculosis; TB, tuberculosis.

Data extracted from the South African National database included patient demographics, previous TB history, drug susceptibility testing results (molecular testing for rifampicin, isoniazid, fluoroquinolones, and second line injectables), sputum smear and culture, the initial patient regimen, subsequent regimen changes, and end of treatment outcomes. From 2019, drug-associated adverse events were reported. Data were retrieved for: (i) 1 January 2017 to 31 December 2017, for the ethionamide-containing regimen, and (ii) from 1 January 2019 to 31 December 2019, for the linezolid-containing regimen. The ethionamide regimen was implemented progressively over 2017, which resulted in the exclusion of 2085 (59%) patients from the 2017 data, as they received an older regimen which included injectables (figure 1).

### Outcome Definitions

Treatment outcomes were categorised according to the South African TB program's classification [5], which aligned with WHO definitions [15].

Treatment outcomes were determined to 24 months using data from EDRWeb. Mortality up to 24 months post treatment initiation was derived from the South African national death registry, a universal registry, which could be linked to the EDRWeb data set using the national ID number. Individuals who transferred out or had undefined outcomes were excluded from the analysis. Treatment success was defined as treatment completion or microbiological cure (culture conversion) between 250 and 365 days after treatment initiation, without subsequent relapse or death. Individuals reported to have died between enrollment, and 24 months were assigned the outcome “death.” Individuals with a second notified TB episode occurring before 24 months were classified as “relapse.” Other outcomes included treatment failure and loss to follow-up.

### Adverse Event Reporting

Data on adverse events were available for the linezolid-containing regimen only. Within the South African TB program only adverse events of grade 3 and above were reported. Data for permanent discontinuations of linezolid were available; dose changes were not reported. Adverse events were reported by local clinicians according to the Common Terminology Criteria for Adverse Events (CTCAE) v 5.0 [16]. (for adverse event monitoring see Supplementary Appendix p3). Adverse events of special interest pre-specified by the WHO Guideline Development Group were reported: hepatitis, myelosuppression, optic neuritis, prolonged QTc, and peripheral neuropathy.

### Statistical Methods

The primary analysis compared 24-month outcomes between patients receiving the ethionamide regimen and those receiving the linezolid regimen. Inverse probability of treatment weighting (IPTW) was used to control for confounding between groups. Covariates included in propensity score estimation were selected a-priori, based on established knowledge of confounding factors in outcomes, including age, sex, human immunodeficiency virus (HIV) status, antiretroviral therapy (ART) for patients with positive HIV status, acid fast bacilli (AFB) smear at baseline, and previous TB treatment with first line drugs. Weighted samples were used in a log-binomial regression model to assess for an association between primary outcomes and treatment regimen, with measure of effect presented as an adjusted relative risk. Standardized mean difference was computed to ensure balance of IPTW distribution across cohorts, with a value of <10% accepted.

The proportion of missing data ranged from 0.1% to 10% across variables included in propensity score analysis (Table 1). Missing data affecting potential confounders were dealt with using multiple imputation by chain equations (MICE) (for detailed statistical methods see Supplementary Appendix p2).

**Table 1.** Patient Demographics and Clinical Outcomes of Patients Treated in the Ethionamide-containing Group and the Linezolid-containing Group

	Linezolid-containing Group n (%)	Ethionamide-containing Group n (%)
Characteristics		
Total	4244 (100)	817 (100)
Age, years (mean, SD)	38 (13)	42 (14)
Sex		
Male	2536 (60)	494 (60)
Female	1708 (40)	323 (40)
HIV status		
Positive	2826 (67)	589 (72)
Negative	1406 (33)	227 (28)
Missing	12 (0.2)	1 (0.1)
ART status among PWH		
On ART	2603/2826 (92)	567/589 (96)
Not on ART	44/2826 (2)	5/589 (1)
Missing	179/2826 (6)	18/589 (3)
Previous treatment for TB with first line drugs		
Yes	1654 (39)	347 (42)
No	2590 (61)	470 (58)
Disease site		
Pulmonary only	4125 (97)	797 (98)
Extrapulmonary	81 (2)	20 (2)
AFB smear at baseline		
Positive	1451 (34)	318 (39)
Negative	2379 (56)	420 (51)
Missing	414 (10)	79 (10)
Sputum culture at baseline		
Positive	2101 (50)	355 (43)
Negative	875 (21)	166 (20)
Missing	1268 (30)	296 (36)
Isoniazid resistance status		
Sensitive	1421 (33)	328 (40)
Resistant	1706 (40)	300 (37)
Missing	1117 (26)	189 (23)
Drugs included in treatment regimen^a^		
Bedaquiline	4244 (100)	880 (100)
Linezolid	4244 (100)	53 (6)
Moxifloxacin/Levofloxacin	4244 (100)	806 (99)
Ethionamide^b^	299 (7)	817 (100)
Ethambutol	4155 (98)	789 (97)
Pyrazinamide	4170 (98)	812 (99)
Isoniazid	4073 (96)	817 (100)
Clofazamine	4098 (97)	791 (97)
Clinical outcomes 24 m post commencement of treatment^c^		
Treatment success^d^	2705 (64)	538 (66)
Treatment failure	47 (1)	10 (1)
Death	853 (20)	176 (22)
Lost to follow-up	639 (15)	93 (11)

Abbreviations: AFB, acid fast bacilli; ART, antiretroviral therapy; HIV, human immunodeficiency virus; PWH, people with HIV; TB, tuberculosis.

^a^This includes drugs used at any point during the regimen.

^b^For the purposes of the primary analysis patients receiving any ethionamide in the 2019 regimen and those receiving any linezolid in the 2017 regimen were not excluded. A sensitivity analysis excluding people receiving ethionamide in 2019 and linezolid in 2017 was performed and is included in the Supplementary appendix (Supplementary Table 3). There was no significant impact of their exclusion on the comparative analyses.

^c^Deaths reported in the post treatment period up to 24 m after commencement of treatment were classified death, regardless of prior TB recurrence.

^d^Includes cure and treatment completion.

These analyses were repeated for subgroup populations: age, people with HIV (PWH), AFB smear status at baseline, previous TB treatment, and isoniazid resistance.

In secondary analysis, a Cox proportional hazards model was fitted to identify associations between patient characteristics and mortality. Time zero was taken as start of treatment and patients were followed until time of death or censored at 24 months. Patients lost to follow-up were censored at last recorded treatment date. Percentage survival at 12 months and 24 months was calculated with 95% confidence intervals (CI).

Time to sputum culture conversion (SCC) was compared between groups. SCC was defined as time from start of treatment to time of 2 consecutive negative sputum samples taken more than 30 days apart. The outcome of interest was SCC within 365 days. Time zero was start of treatment and patients were followed until culture conversion or were censored at 365 days. Patients whose baseline sputum status was negative or unknown were excluded. Patients lost to follow up were censored at date of last treatment. Those who died were censored at date of death. Time to SCC was compared between treatment groups using a Cox proportional hazards model. Median time to SCC was reported with interquartile range (IQR).

Variables included in Cox regression models were age, sex, treatment regimen, HIV status, ART status, AFB smear status at baseline and previous TB treatment with first line drugs. All model assumptions were checked including linearity of continuous variables and proportional hazards assumptions using Schoenfeld residuals plots.

Statistical analyses were performed in R: A language and environment for statistical computing, version 4.0.4.

### Ethical Approval

Ethical approval was granted by Human Research Ethics Committee, Medical University of Witwatersrand (Johannesburg, South Africa; #M210979).

## RESULTS

### Demographics and Clinical Characteristics

A total of 9259 patients commenced a shorter regimen for treatment of MDR/RR-TB, comprising 3517 in 2017 and 5742 in 2019. Figure 1 shows exclusion of patients who did not meet eligibility criteria for the 9-month regimen according to South African protocol guidelines or eligibility criteria for this analysis. The total number of patients receiving the ethionamide-containing regimen was 817, and 4244 received the linezolid-containing regimen (Figure 1). Baseline characteristics are shown in Table 1.

### Comparison of 2017 Ethionamide and 2019 Linezolid-containing Regimens

At 24 months post treatment commencement, no evidence of a difference was observed in the proportion of successful outcomes between treatment groups (adjusted relative risk [aRR] = 0.96, 95% CI .91, 1.01, *P* = .14) (Table 2) or in the proportion of patients surviving to 24 months (aRR = 1.01, 95% CI .87, 1.17, *P* = .92). A higher proportion of patients receiving linezolid were lost to follow-up (aRR = 1.22, 95% CI .99, 1.50, *P* = .06); however, the estimates were imprecise and should be interpreted with caution. The point estimate for treatment failure was 13% lower in the linezolid group; however, wide confidence intervals crossed the null (aRR = 0.87, 95% CI .44, 1.75, *P* = .70) (Table 2). Subgroup stratification by age, sex, HIV status, AFB smear status at baseline, previous history of TB, and isoniazid resistance status showed similar results. However, risk of loss to follow-up was higher in the linezolid group for both HIV negative individuals (aRR = 1.63, 95% CI 1.04, 2.54, *P* = .03) and those who previously received treatment for TB (aRR = 1.36, 95% CI 1.00, 1.86, *P* = .05) (Table 2).

**Table 2.** Primary Analysis Comparing Outcomes Among Patients Treated in the Linezolid-containing Group With the Ethionamide-containing Group

Outcome			Risk of Treatment Outcome in the Linezolid Group Compared With the Ethionamide Group		
	Linezolid- containing Group	Ethionamide- containing Group	aRR	95% CI	*P* Value
	Events/Total (%)	Events/Total (%)			
Total population					
Success vs all other outcomes	2705/4244 (64)	538/817 (66)	0.96	(.91, 1.01)	.14
Treatment failure or recurrence versus all other outcomes	47/4244 (11)	10/817 (12)	0.87	(.44, 1.75)	.70
Death vs survival	853/4244 (20)	176/817 (22)	1.01	(.87, 1.17)	.92
Lost to follow up vs all other outcomes	639/4244 (15)	93/817 (11)	1.22	(.99, 1.50)	.06
PWH					
Success vs all other outcomes	1759/2826 (62)	380/589 (65)	0.97	(.91, 1.04)	.38
Treatment failure or recurrence vs all other outcomes	25/2826 (1)	8/589 (1)	0.62	(.28, 1.39)	.25
Death vs survival	629/2826 (22)	129/589 (22)	1.05	(.88, 1.24)	.59
Lost to follow-up vs all other outcomes	413/2826 (15)	72/589 (12)	1.11	(.88, 1.41)	.38
HIV negative					
Success vs all other outcomes	939/1406 (67)	157/227 (69)	0.93	(.85, 1.02)	.1
Treatment failure or recurrence vs all other outcomes^a^	22/1406 (2)	2/227 (1)	1.78	(.42, 7.50)	.56
Death vs survival	221/1406 (16)	47/227 (21)	0.92	(.68, 1.24)	.57
Lost to follow-up vs all other outcomes	224/1406 (16)	21/227 (9)	1.63	(1.04, 2.54)	.03
AFB smear positive at baseline					
Success vs all other outcomes	931/1451 (64)	204/318 (64)	0.98	(.90, 1.08)	.72
Treatment failure or recurrence vs all other outcomes^a^	23/1451 (2)	4/318 (1)	1.26	(.44, 3.62)	.80
Death vs survival	278/1451 (19)	76/318 (24)	0.88	(.70, 1.10)	.26
Lost to follow-up vs all other outcomes	219/1451 (15)	34/318 (11)	1.32	(.93, 1.86)	.12
AFB smear negative at baseline					
Success vs all other outcomes	1608/2379 (68)	293/420 (70)	0.97	(.9, 1.04)	.43
Treatment failure or recurrence vs all other outcomes	18/2379 (1)	5/420 (1)	0.58	(.21, 1.60)	.30
Death vs survival	392/2379 (16)	77/420 (18)	0.95	(.75, 1.19)	.64
Lost to follow-up vs all other outcomes	361/2379 (15)	45/420 (11)	1.3	(.97, 1.76)	.08
Previous treatment for TB					
Success vs all other outcomes	964/1654 (58)	213/347 (61)	0.95	(.87, 1.05)	.34
Treatment failure or recurrence vs all other outcomes	25/1654 (2)	7/347 (2)	0.73	(.31, 1.69)	.46
Death vs survival	377/1654 (23)	86/347 (25)	0.95	(.77, 1.17)	.64
Lost to follow-up vs all other outcomes	288/1654 (17)	41/347 (12)	1.36	(1.00, 1.86)	.05
No previous treatment for TB					
Success vs all other outcomes	1741/2590 (67)	325/470 (69)	0.96	(.90, 1.03)	.26
Treatment failure or recurrence vs all other outcomes^a^	22/2590 (1)	3/470 (1)	1.33	(.40, 4.43)	1.00
Death vs survival	476/2590 (18)	90/470 (19)	1.06	(.86, 1.31)	.60
Lost to follow-up vs all other outcomes	351/2590 (14)	52/470 (11)	1.12	(.84, 1.48)	.44
Isoniazid resistant^b^					
Success vs all other outcomes	1117/1706 (65)	215/300 (72)	0.9	(.83, .97)	.01
Treatment failure or recurrence vs all other outcomes	31/1706 (2)	5/300 (2)	1.09	(.42, 2.83)	.86
Death vs survival	296/1706 (17)	49/300 (16)	1.21	(.91, 1.62)	.19
Lost to follow-up vs all other outcomes	262/1706 (15)	31/300 (10)	1.38	(.97, 1.97)	.08
Isoniazid sensitive^c^					
Success vs all other outcomes	930/1421 (65)	206/328 (62)	1.04	(.95, 1.14)	.41
Treatment failure or recurrence vs all other outcomes^a^	13/1421 (1)	3/328 (1)	1.00	(.29, 3.49)	1.00
Death vs survival	269/1421 (19)	82/328 (25)	0.8	(.64, .99)	.04
Lost to follow-up vs all other outcomes	209/1421 (15)	37/328 (11)	1.23	(.88, 1.71)	.23

RR is presented as an adjusted figure following inverse probability of treatment weighting using the propensity score, if less than 5 events occurred in one or both cohorts, outcome is presented as an unadjusted relative risk.

Abbreviations: aRR, adjusted relative risk; AFB, acid fast bacilli; CI, confidence interval; HIV, human immunodeficiency virus; PWH, people with HIV; TB, tuberculosis.

^a^Denotes outcome presented as unadjusted relative risk.

^b^Isoniazid resistance diagnosed on both genotypic and phenotypic testing results.

^c^Isoniazid sensitivity only diagnosed on genotypic results; no phenotypic results available.

For patients with isoniazid resistance at treatment initiation, probability of success was lower among those receiving the linezolid-containing regimen (aRR = 0.9, 95% CI .83, .97, *P* = .01) compared to the ethionamide-containing regimen. Mortality was lower with the linezolid-containing regimen (aRR = 0.80, 95% CI .64, .99, *P* = .04) for those with isoniazid-susceptible disease (Table 2).

### Predictors of Mortality

A total of 1028 deaths occurred within 24 months of treatment initiation in the 2 groups, with 176/817 (22%) deaths in the ethionamide-containing regimen, and 853/4244 (20%) deaths in the linezolid-containing regimen (Table 2), In addition, 14% were censored due to loss to follow-up. Probability of survival to 12 months was 81% (95% CI .79, .82), and probability of survival to 24 months was 78% (95% CI .77, .80), indicating most deaths occurred within the first 12 months. No difference was observed in survival time between treatment groups to 24 months (HR = 1.03, 95% CI .86, 1.23, *P* value = .76). Increasing age, positive AFB status at baseline, positive HIV status, lack of ART treatment, and previous treatment for TB with first line drugs were associated with reduced survival time to 24 months (Table 3).

**Table 3.** Multivariable Cox Regression for Time to Death From Treatment Commencement Until 24 Months

Variable	Hazard Ratio of Death	95% CI	*P* Value
Sex			
Male	Ref		
Female	0.98	(.85, 1.14)	.83
Age, years	1.04	(1.03, 1.05)	<.0001
Treatment cohort			
2017 Ethionamide group	Ref		
2019 Linezolid group	1.02	(.86, 1.23)	.76
HIV status			
HIV negative	Ref		
HIV positive on ART	1.48	(1.26, 1.75)	
HIV positive no ART	7.51	(4.79, 11.79)	<.0001*
AFB Smear status at baseline			
Negative	Ref		
Positive	1.30	(1.13, 1.51)	.0003
Previous first line treatment for TB			
No	Ref		
Yes	1.22	(1.05, 1.40)	.007

Abbreviation: AFB, acid fast bacilli; ART, antiretroviral therapy; CI, confidence interval; HIV, human immunodeficiency virus; TB, tuberculosis.

^*^Estimated using Wald test.

### Bacteriological Outcomes

Among 2300 patients across treatment groups with a positive culture at baseline, 1900 (83%) achieved culture conversion within 12 months. Among 400 patients censored prior to completing treatment, 40% were lost to follow-up, 52% died prior to SCC, and 8% had not achieved culture conversion by 12 months. Median time to conversion in the ethionamide and linezolid groups was 60 days (IQR 34–94) and 57 days (IQR 41–87), respectively. After adjustment using Cox regression, AFB status at baseline was found to violate the proportional hazard assumption; the model was stratified by AFB status to deal with this deviation. In the final model no difference was observed in time to culture conversion for the ethionamide-containing regimen compared to the linezolid-containing regimen (HR = 1.04, 95% CI .91, 1.17, *P* = .58).

### Adverse Events

In the linezolid group, grade 3 and 4 adverse events were reported in 213 patients (5%) (Table 4). The most reported severe adverse event was myelosuppression (2%). There were 90 (2%) permanent discontinuations of linezolid and 10 (0.2%) of bedaquiline.

**Table 4.** Grade 3 and Above Adverse Events Reported for All Drugs Used in the Linezolid-containing Group

Characteristic	All Adverse Events Grade 3 and Above	Adverse Events of Special Interest^a,b^				
		Hepatitis	Myelosuppression	Peripheral Neuropathy	Optic Neuritis	QTc Prolongation
	n (%)	n (%)	n (%)	n (%)	n (%)	
Total population (n = 4244)						
≥1 adverse event grade 3 and above^c^	213 (5)	23 (0.5)	88 (2)	13 (0.3)	12 (0.3)	16 (0.4)
Age group						
<15 (n = 69)	3 (4)	1 (1)	1 (1)	0 (0)	0 (0)	0 (0)
15–30 (n = 996)	44 (4)	7 (0.7)	18 (2)	5 (0.5)	2 (0.2)	1 (0.1)
30–50 (n = 2373)	109 (5)	14 (0.6)	52 (2)	4 (0.2)	7 (0.3)	9 (0.4)
50–65 (n = 654)	46 (7)	1 (0.2)	14 (2)	4 (0.6)	1 (0.2)	6 (1)
≥65 (n = 152)	11 (7)	0 (0)	3 (2)	0 (0)	2 (1)	0 (0)
HIV status^d^						
Negative (n = 1406)	60 (4)	9 (1)	15 (1)	4 (0.3)	5 (0.4)	6 (0.4)
Positive (n = 2826)	153 (5)	14 (0.5)	73 (3)	9 (0.3)	7 (0.2)	10 (0.4)

Adverse events are recorded as number of patients experiencing at least 1 adverse event during treatment.

Abbreviation: HIV, human immunodeficiency virus.

^a^Adverse events of special interest which had been pre-specified by the WHO Guideline Development Group included: hepatitis, myelosuppression, peripheral neuropathy, optic neuritis, and prolonged QTc.

^b^The highest grade of adverse event was recorded for patients experiencing at least 1 event during treatment.

^c^Grade 1 and 2 adverse events are not routinely reported in the TB program, facilities are only required to report grade 3 and above events.

^d^12 patients had missing HIV status, however no adverse events were recorded for these patients.

## DISCUSSION

This retrospective cohort study among 5124 patients treated for MDR/RR-TB in South Africa compared outcomes among patients given a 9-month all-oral regimen containing ethionamide with a regimen including linezolid. No evidence of a difference was observed between the two regimens at 24 months for treatment success, treatment failure, survival and time to culture conversion. Loss to follow-up was more common in the linezolid group, and treatment failure less likely to occur, however, the precision for these estimates was low, precluding certainty in the findings.

Observational data from other settings suggests bedaquiline [3, 6, 17–19] and linezolid [6, 18] are associated with improved treatment outcomes for drug-resistant TB compared to earlier regimens. However, data regarding outcomes with regimens containing linezolid mostly involves longer treatment regimens (18–24 months) [6] or small patient cohorts [18]. This study looked at patients taking two-months of linezolid as a part of a 9-month standardised regimen, in a large national programmatic dataset. We identified a 66% success rate using ethionamide in a 9-month regimen, and 64% when linezolid replaced ethionamide, without demonstrating superiority of either regimen.

There are potential benefits conferred by including linezolid in a regimen to treat MDR/RR-TB. Acquired resistance to bedaquiline has been reported, including in South Africa [20]. Therefore, ensuring effective companion drugs are included throughout therapy is essential to preserving existing drugs. Treatment adherence is a significant challenge for patients taking MDR/RR-TB therapy due to the high pill burden [21]. Although reduction in treatment time from 18–24 months to 9 months reduces this burden, the daily number of tablets remains substantial. Treatment with linezolid for two months instead of 4 months with ethionamide, has the advantage of further reducing the total pill burden.

Our study observed high dropout rates with both regimens; this high rate of loss to follow-up has been seen in other studies in similar populations [3, 10]. The use of shorter treatment regimens is suggested to improve adherence [22]. However, rates in this study remain similar to those seen in longer treatments within South Africa [19]. Loss to follow-up was higher in the linezolid regimen (15% vs 11%, RR 1.22 95% CI .99, 1.50), although not reaching the threshold for statistical significance. Reasons for this difference are not certain. Adverse events related to linezolid toxicity, resulting in poorer adherence may have contributed. However, a lack of national reporting of adverse events precluded comparisons of toxicity between the two cohorts. We found no evidence that measured patient characteristics shown to be associated with dropout from MDR/RR-TB treatment such as HIV, previous TB treatment, sex, and age [23], were responsible for the difference by accounting for them in the analysis. However, it is possible other unmeasured factors may be responsible. It is unlikely that differences in mortality were responsible, given the high uptake of death notifications in South Africa, which were linked to our cohort. Mortality was well reported, and reports of mortality were balanced between groups. It is possible there were differences in programmatic support for patients. This ethionamide group may have been more closely monitored by the program, because the 2017 ethionamide-containing cohort was the first regimen to be “injectable-free”. Nonetheless, loss to follow-up is an important concern with both regimens, indicating the importance of adherence support to ensure patients complete treatment.

Two-year mortality was substantial within both cohorts, 22% and 20% in the ethionamide and linezolid cohort, respectively. This may be reflective of a population with high rates of comorbidity, including HIV and high rates of previous TB treatment, both factors associated with poorer outcomes and challenges with regimen adherence [24]. It is important to note it is not possible to distinguish mortality due to treatment from other causes within this data set.

A lower incidence of adverse events was reported for the linezolid-containing regimen in this study compared to recent clinical trials [11, 25]. However, underreporting is more likely in programmatic settings where toxicity is unmonitored as intensively as within a clinical trial setting. Programmatic use of linezolid within national TB programs is challenging owing to managing toxic side effects in resource-limited settings [9, 26, 27]. Nevertheless, our study showed that the majority of patients receiving linezolid were able to successfully complete treatment. However, less toxic alternatives to linezolid are urgently needed to reduce participant loss to follow-up and toxicity. Robust prospective monitoring of all patients with MDR-TB will be required to optimise individual patient care and ensure programmatic outcomes are optimal [28].

This study has several limitations. Selection bias may have occurred if the demographics and other clinical factors related to the outcomes changed over time. Clinical and geographical factors were adjusted for in the statistical analysis; however, this is programmatic data, and so not all patient characteristics determining treatment choice were captured, such as indicators of disease severity. Therefore, unmeasured confounding may have led to bias between groups. Generalisability of these findings to patients with more severe disease may be limited, as selection protocols for these South African regimens only include those with pulmonary disease without extensive cavitation, non-complicated extra pulmonary disease and those without advanced drug resistance. Detailed information on some outcomes ( unreported relapse, acquired drug-resistance, adverse events) were also incomplete, preventing direct comparison between regimens.

Strengths of this study include the analysis of a large population from a programmatic cohort, representing current practice within the South African TB Program. This analysis justifies the scale-up of linezolid-containing regimens. In addition, accurate recording of mortality during and post-treatment, even among those lost to follow-up, was also a strength due to linkage to the national death registry. Finally, multiple imputation and propensity score analysis mitigated the impact of missing data and potential confounders.

This study has important implications for global TB control policy. A 9-month regimen containing either linezolid for 2 months or ethionamide for 4 months can be considered equally appropriate and has been recommended in updated 2022 WHO guidelines. This recommendation applies to those with MDR/RR-TB in whom resistance to fluoroquinolones has been excluded and for those who are not eligible to receive a 6-month pretomanid-containing regimen (bedaquiline, pretomanid, linezolid and moxifloxacin [BPaLM] or bedaquiline, pretomanid, and linezolid [BPaL]) [2].

In conclusion, this retrospective cohort study found similar outcomes for patients receiving regimens containing linezolid for 2 months or ethionamide for 4 months as a part of a standardized 9-month regimen. Further programmatic research is required to explore the safety of these regimens in a broad range of settings. Less toxic drugs for treating MDR-TB are urgently needed.

## Supplementary Data

Supplementary materials are available at *Clinical Infectious Diseases* online. Consisting of data provided by the authors to benefit the reader, the posted materials are not copyedited and are the sole responsibility of the authors, so questions or comments should be addressed to the corresponding author.

## Supplementary Material

ciae145_Supplementary_Data

## Notes

***Author Contributions.*** H. M.: conceptualization, data curation, formal analysis, methodology, writing—original draft. N. N.: data curation, methodology, writing- review and editing. T. H.: data curation, writing—review and editing. Medea Gegia: supervision, writing—review and editing. F. M.: supervision, writing—review and editing. L. N.: supervision, writing—review and editing. Samuel Schumacher: supervision, writing—review and editing. T. S.: supervision, methodology, formal analysis, writing—review and editing. K, N.: supervision, writing—review and editing. G. F.: conceptualization, supervision, writing—review and editing.

***Acknowledgments.*** The authors acknowledge the contribution of the South African National Tuberculosis Program to provide data for these cohorts. The support of the World Health Organization secretariat is also acknowledged. We acknowledge contributions of Jonathon Campbell at McGill University to data preparation.

***Data sharing agreement.*** Deidentified participant data and data dictionary will be publicly available on the WHO Data Repository, hosted at the University College London.

***Financial support*.** This work was supported by funding provided by the World Health Organization. G. J. F. was supported by an Australian National Health and medical Research council Investigator Grant (APP2007920).
