# Supplementary material for: Treatment of Multidrug-resistant or Rifampicin-resistant Tuberculosis With an All-oral 9-month Regimen Containing Linezolid or Ethionamide in South Africa: A Retrospective Cohort Study
Source: Clin Infect Dis. 2024 Mar 25;78(6):1698–706. doi: 10.1093/cid/ciae145 (PMC11175697; doi:10.1093/cid/ciae145)
Supplement: ciae145_Supplementary_Data [file ciae145_supplementary_data.docx]

**Supplementary material to: Treatment of multidrug-resistant or rifampicin-resistant tuberculosis with an all-oral, 9-month regimen containing linezolid or ethionamide in South Africa: a retrospective cohort study**

Hannah Morgan, Norbert Ndjeka, Tasnim Hasan, Medea Gegia, Fuad Mirzayev, Linh N. Nguyen, Samuel Schumacher, Timothy E Schlub, Kogieleum Naidoo, Greg Fox

**Contents**

Supplementary methods ………………...………………………………………………………………………...2

Supplementary Table S1: Inclusion and exclusion criteria for the 9-month regimen as defined in the South Africa National Tuberculosis program.……………...…………………………………………………………….4

Supplementary Table S2: Distribution of patients treated with short all-oral regimen by province of treatment...5

Supplementary Table S3: Primary analysis outcomes when excluding patients with any use of linezolid from the ethionamide group and patients with any use of ethionamide from the linezolid group ……...………………….6

Supplementary Table S4: Fluoroquinolone resistance status as recorded in EDRweb across treatment groups….7

Supplementary Table S5: Primary analysis outcomes excluding any patients with missing data for fluoroquinolone resistance status………………………………………………………………………………….8

Supplementary Table S6: Comparison of primary outcomes using multivariable analysis and a mixed effects logistic analysis including province as a random effect..………………………………………………………….9

Supplementary Table S7: Primary analysis outcomes comparing linezolid and ethionamide group stratified by age group…………………………………………………………………………………………………….…...10

Supplementary Table S8: Primary analysis outcomes comparing patients treated using a short all-oral regimen without linezolid to patients treated with a short all-oral regimen including linezolid, as presented to the guideline development group for WHO guideline review process………………………………….…………...11

Supplementary Figure S1: Kaplan Meier survival estimate for time to loss to follow up by treatment group…..13

**Supplementary Methods**

**Description of drug regimens**

The all-oral regimen used in South Africa in 2017 involved the initiation of bedaquiline, levofloxacin/moxifloxacin, clofazimine, ethionamide, ethambutol, isoniazid (high dose) and pyrazinamide. All seven drugs were given for 4 months, with the possibility of extending to 6 months if the patient’s sputum remained bacteriologically positive at the end of the fourth month. Ethionamide and high-dose isoniazid were dropped after 4 or 6 months, depending on the decision to extend treatment based on smear status at month 4. This was followed by 5 months of treatment with levofloxacin/moxifloxacin, clofazimine, ethambutol and pyrazinamide. Bedaquiline was given for 6 months but could be extended to 9 months, particularly if the initial phase was extended from 4 to 6 months due to a lack of sputum conversion at month 4. The all-oral regimen used in 2019 was identical to the ethionamide-containing regimen, except ethionamide was replaced by linezolid (600mg daily) for the first two months of the regimen. All other drugs and dosing remained the same^1^.

**Dosages for shorter 9-month treatment regimens**

The seven-drug all-oral ethionamide-containing regimen, used in South Africa in 2017 included 9-11 months of; levofloxacin (15-20mg/kg, daily, maximum 1000mg) or moxifloxacin (7·5-10mg/kg, daily, maximum 800mg), clofazimine (2-3mg/kg, daily, maximum 100mg), ethambutol (15-20mg/kg, daily, maximum 1200mg), and pyrazinamide (30-40mg/kg, daily, maximum 2000mg). This was supplemented by 4-6 months of ethionamide (15-20mg/kg, daily) and high dose isoniazid (10 mg/kg, daily, maximum 600mg, except when fully susceptible to isoniazid, in which case 5mg/kg was given) and bedaquiline (400mg, once daily for 2 weeks, then 200mg three times per week for 22 week) for 6 months, which could be extended to 9 months^1^.

The seven-drug all-oral regimen, used in 2019, was identical to the ethionamide-containing regimen except ethionamide was replaced by linezolid (600mg daily) for the first two months of the regimen.

**Monitoring of adverse events**

Whilst the patient was receiving linezolid, full blood count and neutrophil count were done before starting treatment, at week 2 and week 4, and then monthly until linezolid was stopped. Vision was assessed at baseline and then monthly through clinical questioning and use of Snellen chart whilst on linezolid. ECG was performed at baseline and repeated monthly whilst on bedaquiline^1^.

**Statistical analysis**

The primary analysis compared 24-month outcomes between patients receiving the ethionamide regimen and those receiving the bedaquiline regimen. Inverse probability of treatment weighting (IPTW) was used to control for confounding between the study groups. Propensity scores were estimated using logistic regression as the probability of receiving linezolid conditional on baselines characteristics. Covariates for inclusion in propensity score estimation were selected a-priori, based on established knowledge of confounding factors in outcomes, these included age, sex, HIV status, antiretroviral therapy (ART) for patients with positive HIV status, acid fast bacilli (AFB) smear at baseline and previous TB treatment with first line drugs. Individuals were weighted using the inverse of the probability of treatment, creating a pseudo-population in which treatment assignment was independent of measured baseline characteristics^2^. Weighted samples were used in a log-binomial regression model to assess for an association between primary outcomes and treatment regimen, with measure of effect presented as an adjusted relative risk. Where event rates were close to the boundary (fewer than five positive or negative events), an unadjusted relative risk was calculated. For outcomes where number of events was zero, unadjusted risk difference was calculated. Standardised mean difference was computed to ensure balance of IPTW distribution across cohorts, with a value of <10% accepted.

The proportion of missing data ranged from 0.1 to 10% across the variables included in propensity score analysis (table 1). Missing data affecting potential confounders were dealt with using multiple imputation by chain equations (MICE)^3^, allowing imputation across multiple variables, with 10 iterations per variable and 40 imputed data sets. This was carried out using R: library(mice)^4^. All covariates included in propensity score estimation, treatment and outcome were used to support the imputation process. For use of imputed data with the propensity score, the within approach was used for combining imputed datasets and propensity scores^5^.

**Sensitivity analyses**

As this study was located in a national TB programme, a standardised regimen was used for treatment in the two cohorts. Patients were included in the primary analysis following the principle of intention to treat, even if the regimen they received differed from the standardised treatment protocol. As such, in the primary analysis, 53 (6%) patients in the ethionamide regimen received linezolid during treatment, and 299 (7%) in the linezolid regimen received ethionamide during treatment. A sensitivity analysis was conducted in which patients taking any linezolid in 2017 and those taking any ethionamide in 2019 were removed (see Supplementary Table S2). This sensitivity analysis did not qualitatively change the outcomes reported.

Patients with known fluoroquinolone susceptibility, or a missing fluoroquinolone resistance result, were included in the analysis. Sensitivity analysis that excluded those with missing fluoroquinolone resistance status produced a similar result to the analysis where this group were included (see Supplementary Tables S3 and S4).

Data for both the 2017 and 2019 cohort was collected from 9 provinces across South Africa. To explore whether there was a clustering effect due to the province in which treatment was received, a multivariable logistic model was compared with a mixed logistic model where province was included as a random intercept term. Covariates to be included in the model were determined a- priori, based on availability of data and previous knowledge of potential confounding factors. These were consistent with variables included in the propensity score model. Fixed variables included in both models were treatment regimen, age, sex, HIV status, ART status at baseline, acid fast bacilli (AFB) smear status at baseline and previous TB treatment with first line drugs. This was a complete case analysis; multiple imputation was not used. Linearity assumptions were checked for continuous variables. Results were presented using odds ratio, with 95% CI (Supplementary Table S5). The use of province as a random effect did not change overall study conclusions.

Within the primary analysis the point estimate for loss to follow up versus all other outcomes was higher in the linezolid group than the ethionamide group, with a wide confidence interval associated with the point estimate (15% vs 11%, RR 1.22 95%CI 0.99-1.50). To further analyse the difference in loss to follow up that occurred between the two treatment groups, time to loss to follow up within each treatment group was estimated using Kaplan-Meier curves. For the analysis of loss to follow up, the outcome of interest was loss to follow up to 24 months following treatment commencement. An observation was considered censored if the outcome of interest was not achieved. Observations were censored if the patient had a successful outcome, treatment failure or died.

**Table S1: Inclusion and exclusion criteria for the 9-month regimen as defined in the South Africa National Tuberculosis program^1^**

| **Inclusion criteria for the 9-month regimen** |
| --- |
| 1. Individual with rifampicin resistant (RR)/ multi-drug resistant (MDR)-tuberculosis (TB), without greater than 1-month prior exposure to second line treatment 2. Uncomplicated RR/MDR extra-pulmonary TB 3. People living with HIV already on anti-retroviral therapy (ART) or due to start ART 4. Pregnant women with pulmonary TB with or without uncomplicated extra pulmonary TB may receive the 9-month regimen once the case has been reviewed by the National Clinical Advisory Committee (NCAC) for surveillance purposes, due to limited data on use of these regimens in pregnant women |
| **Exclusion Criteria for the 9-month regimen** |
| 1. Any previous exposure to second line treatment for more than 1 month 2. Resistance to fluroquinolones or injectable agents 3. Both isoniazid mutations (inhA and katG) on line probe assay (LPA) 4. RR/MDR-TB with additional resistance to bedaquiline, linezolid or ceftazidime 5. RR/MDR-TB in cases where additional second line resistance is suspected, despite confirmed susceptibility on 2^nd^ line LPA or phenotypic DST; this includes:  - Close contacts of patients with confirmed pre-extensively drug resistant-TB or extensively drug resistant-TB - Close contacts of patients with second line TB treatment failure  1. Complicated and/or severe forms of extra-pulmonary RR/MDR-TB disease including meningitis, osteo-articular, pericardial effusion, abdominal TB 2. RR/MDR-TB with extensive pulmonary disease, including extensive bilateral pulmonary cavitations |

**Table S2: Distribution of patients treated with short all-oral regimen by province of treatment**

|  | **Linezolid group n (%)** | | | | | **Ethionamide group n (%)** | | | | |
| --- | --- | --- | --- | --- | --- | --- | --- | --- | --- | --- |
| **Province** | **Total**  **Cases** | **Success** | **Failure** | **Death** | **LTFU** | **Total Cases** | **Success** | **Failure** | **Death** | **LTFU** |
| Eastern Cape | 836 (20) | 541 (65) | 8 (1) | 168 (20) | 119 (14) | 236 (28) | 168 (71) | 5 (2) | 44 (19) | 19 (8) |
| Free State | 141 (3) | 97 (69) | 1 (1) | 24 (17) | 19 (13) | 37 (5) | 26 (70) | 0 (0) | 8 (22) | 3 (8) |
| Gauteng | 494 (12) | 303 (61) | 7 (1) | 101 (20) | 83 (17) | 92 (11) | 47 (51) | 0 (0) | 21 (23) | 24 (26) |
| Kwazulu-Natal | 1076 (25) | 688 (64) | 11 (1) | 216 (20) | 161 (15) | 197 (24) | 137 (70) | 1 (1) | 35 (18) | 24 (12) |
| Limpopo | 122 (3) | 71 (58) | 2 (2) | 27 (22) | 22 (18) | 24 (3) | 16 (67) | 0 (0) | 7 (29) | 1 (4) |
| Mpumalanga | 255 (6) | 169 (66) | 4 (2) | 48 (18) | 34 (13) | 112 (14) | 79 (70) | 2 (2) | 20 (18) | 11 (10) |
| North West | 186 (4) | 126 (68) | 2 (1) | 25 (13) | 33 (17) | 79 (10) | 40 (51) | 1 (1) | 27 (34) | 11 (14) |
| Northern Cape | 142 (3) | 88 (62) | 1 (1) | 36 (25) | 17 (11) | 28 (3) | 15 (54) | 0 (0) | 13 (46) | 0 (0) |
| Western Cape | 992 (23) | 622 (63) | 11 (1) | 208 (21) | 151 (15) | 12 (1) | 10 (83) | 1 (8) | 1 (8) | 0 (0) |

LTFU=loss to follow up

**Table S3: Primary analysis outcomes when excluding patients with any use of linezolid from the ethionamide group and patients with any use of ethionamide from the linezolid group**

|  |  |  | **Risk of treatment outcome in the linezolid group compared with the ethionamide group** | | | | |
| --- | --- | --- | --- | --- | --- | --- | --- |
|  | **Linezolid group** | **Ethionamide group** | **Unadj. RR** | **95% CI** | **aRR** | **95% CI** | **p value** |
| **Outcome** | **Events/Total** | **Events/Total** |  |  |  |  |  |
|  |  |  |  |  |  |  |  |
| **All Cases** |  |  |  |  |  |  |  |
| Success vs all other outcomes | 2532/3945 | 506/764 | 0.97 | (0.92, 1.02) | 0.96 | (0.91, 1.02) | 0.18 |
| Failure or recurrence versus all other outcomes | 46/3945 | 9/764 | 0.99 | (0.49, 2.01) | 0.93 | (0.45, 1.91) | 0.83 |
| Death vs survival | 775/3945 | 164/764 | 0.92 | (0.79, 1.06) | 0.98 | (0.84, 1.15) | 0.81 |
| Lost to follow up vs all other outcomes | 592/3945 | 85/764 | 1.35 | (1.09, 1.67) | 1.26 | (1.01, 1.56) | 0.038 |
| **Subgroup analyses for outcomes success vs all other outcomes*** |  |  |  |  |  |  |  |
| PLHIV | 1645/2618 | 355/549 | 0.97 | (0.91, 1.04) | 0.98 | (0.91, 1.05) | 0.53 |
|  |  |  |  |  |  |  |  |
| HIV negative | 882/1318 | 150/214 | 0.95 | (0.87, 1.05) | 0.92 | (0.83, 1.01) | 0.077 |
|  |  |  |  |  |  |  |  |
| AFB smear positive at baseline | 867/1318 | 191/293 | 0.99 | (0.90, 1.08) | 0.97 | (0.88, 1.06) | 0.52 |
|  |  |  |  |  |  |  |  |
| AFB smear negative at baseline | 1504/2212 | 278/400 | 0.98 | (0.91, 1.05) | 0.98 | (0.91, 1.06) | 0.62 |
|  |  |  |  |  |  |  |  |
| Previous treatment for TB | 909/1547 | 198/320 | 0.95 | (0.86, 1.04) | 0.96 | (0.87, 1.06) | 0.38 |
|  |  |  |  |  |  |  |  |
| No previous treatment for TB | 1623/2398 | 308/444 | 0.98 | (0.91, 1.04) | 0.96 | (0.90, 1.03) | 0.28 |
|  |  |  |  |  |  |  |  |
| Isoniazid resistant | 1038/1572 | 200/279 | 0.92 | (0.85,1.00) | 0.91 | (0.84, 0.99) | 0.022 |
|  |  |  |  |  |  |  |  |
| Isoniazid sensitive | 878/1347 | 193/305 | 1.0.3 | (0.94,1.13) | 10.2 | (0.93, 1.13) | 0.62 |

Unadj. RR= unadjusted relative risk. aRR=adjusted relative risk. AFB=acid fast bacilli. PLHIV= people living with HIV.

RR is presented as an adjusted figure following inverse probability weighting using the propensity score

***for subgroup analyses only the outcome of success versus all other outcomes was included

In this sensitivity analysis additional exclusion criteria were applied to the data; in the 2017 ethionamide group all patients were excluded who had received any linezolid during treatment, and for the 2019 linezolid group, all patients were excluded who had received any ethionamide during treatment. This resulted in 299 (7%) patients removed from the linezolid group and 53 (6%) from the ethionamide group. Data analysis methods are as used in the primary analysis described in the main manuscript.

**Table S4: Fluoroquinolone resistance status as recorded in EDRweb across treatment groups**

|  | **Linezolid group**  **n (%)** | **Ethionamide group**  **n (%)** |
| --- | --- | --- |
| Fluoroquinolone resistance | 0 | 0 |
| Fluoroquinolone susceptible | 2197 (52) | 400 (49) |
| Fluoroquinolone status missing | 2047 (48) | 417 (51) |

**Table S5: Primary analysis outcomes excluding any patients with missing data for fluoroquinolone resistance status**

|  |  |  | **Risk of treatment outcome in the linezolid group compared with the ethionamide group** | | | | |
| --- | --- | --- | --- | --- | --- | --- | --- |
|  | **Linezolid group** | **Ethionamide group** | **Unadj. RR** | **95% CI** | **aRR** | **95% CI** | **p value** |
| **Outcome** | **Events/Total** | **Events/Total** |  |  |  |  |  |
|  |  |  |  |  |  |  |  |
| **All Cases** |  |  |  |  |  |  |  |
| Success vs all other outcomes | 1433/2197 | 277/400 | 0.94 | (0.8, 1.01) | 0.94 | (0.87, 1.01) | 0.079 |
| Failure or recurrence versus all other outcomes | 30/2197 | 6/400 | 0.91 | (0.38, 2.17) | 0.88 | (0.36, 2.14) | 0.78 |
| Death vs survival | 403/2197 | 73/400 | 1.01 | (0.80, 1.26) | 1.08 | (0.86, 1.37) | 0.50 |
| Lost to follow up vs all other outcomes | 331/2197 | 44/400 | 1.37 | (1.02, 1.84) | 1.28 | (0.90, 1.73) | 0.12 |
| **Subgroup analyses for outcomes success vs all other outcomes*** |  |  |  |  |  |  |  |
| PLHIV | 918/1441 | 194/286 | 0.94 | (0.86, 1.03) | 0.95 | (0.86, 1.04) | 0.23 |
|  |  |  |  |  |  |  |  |
| HIV negative | 514/754 | 83 /114 | 0.94 | (0.83, 1.06) | 0.92 | (0.81, 1.05) | 0.22 |
|  |  |  |  |  |  |  |  |
| AFB smear positive at baseline | 648/982 | 147/214 | 0.9 | (0.87, 1.06) | 0.94 | (0.85, 1.04) | 0.21 |
|  |  |  |  |  |  |  |  |
| AFB smear negative at baseline | 711/1062 | 111157 | 0.95 | (0.85, 1.06) | 0.98 | (0.87, 1.11) | 0.70 |
|  |  |  |  |  |  |  |  |
| Previous treatment for TB | 535/905 | 112/185 | 0.98 | (0.86, 1.11) | 0.99 | (0.87, 1.13) | 0.90 |
|  |  |  |  |  |  |  |  |
| No previous treatment for TB | 898/1292 | 165/215 | 0.91 | (0.83, 0.98) | 0.90 | (0.83, 0.98) | 0.016 |
|  |  |  |  |  |  |  |  |

aRR=adjusted relative risk. AFB=acid fast bacilli. PLHIV= people living with HIV.

RR is presented as an adjusted figure following inverse probability weighting using the propensity score

***for subgroup analyses only the outcome of success versus all other outcomes was included

Within the EDRweb database, resistance to fluoroquinolones was recorded as positive, negative, or missing. The cases with missing fluoroquinolone status who remained on a short all-oral course for the duration of treatment were included in the primary analysis. A sensitivity analysis was carried out removing all patients with missing fluoroquinolone resistance data. Data analysis methods are as used in the primary analysis described in the main manuscript.

**Table S6: Comparison of primary outcomes using multivariable analysis and a mixed effects logistic analysis including province as a random effect**

|  |  |  | **Multivariable logistic model** | | | **Mixed logistic model** | | |
| --- | --- | --- | --- | --- | --- | --- | --- | --- |
| **Outcome** | **Regimen** | **Events/total** | **OR** | **95%CI** | **p value** | **OR** | **95% CI** | **p value** |
| Success vs all other outcomes | Ethionamide group | 491/724 | Ref |  |  |  |  |  |
|  | Linezolid group | 2462/3679 | 0.90 | (0.75,1.06) | 0.20 | 0.91 | (0.76,1.08) | 0.29 |
|  |  |  |  |  |  |  |  |  |
| Failure or recurrence versus all other outcomes | Ethionamide group | 9/724 | Ref |  |  | Ref |  |  |
|  | Linezolid group | 40/3679 | 0.84 | (0.42,1.86) | 0.64 | 0.82 | (0.39, 1.84) | 0.60 |
|  |  |  |  |  |  |  |  |  |
| Death vs survival | Ethionamide group | 148/724 | Ref |  |  | Ref |  |  |
|  | Linezolid group | 627/3679 | 0.97 | (0.79,1.19) | 0.74 | 0.98 | (0.80, 1.20) | 0.74 |
|  |  |  |  |  |  |  |  |  |
| Lost to follow up vs all other outcomes | Ethionamide group | 76/724 | Ref |  |  | Ref |  |  |
|  | Linezolid group | 550/3679 | 1.34 | (1.05,1.77) | 0.02 | 1.32 | (1.02, 1.72) | 0.041 |
|  |  |  |  |  |  |  |  |  |

OR=odds ratio.

Data for both the 2017 and 2019 cohort was collected from 9 provinces across South Africa. A multivariable logistic model containing all variables used in the primary analysis propensity score model was compared with a mixed logistic model where province was included as a random effect. Fixed variables included in both models were treatment regimen, age, sex, HIV status, ART status at baseline, acid fast bacilli (AFB) smear status at baseline and previous TB treatment with first line drugs. This was a complete case analysis; multiple imputation was not used. Results are presented using odds ratio, with 95% CI. The results show there was minimal difference in the size of effect estimated for comparison of outcomes between starting regimens in the multivariable and mixed logistic models, therefore province was not adjusted for in the primary propensity score analysis.

**Table S7: Primary analysis outcomes comparing linezolid and ethionamide groups stratified by age group**

|  |  |  | **Risk of treatment outcome in the linezolid group compared with the ethionamide group** | | |
| --- | --- | --- | --- | --- | --- |
|  | **Linezolid group** | **Ethionamide group** | **aRR** | **95% CI** | **p value** |
| **Outcome** | **Events/Total** | **Events/Total** |  |  |  |
| **Age Category (years)** |  |  |  |  |  |
| **<15** |  |  |  |  |  |
| Success vs all other outcomes* | 57/69 | 4/7 | 1.45 | (0.95, 3.32) | 0.13 |
| Failure or recurrence versus all other outcomes* | 1/69 | 1/7 | 0.1 | (0.01, 0.95) | 0.18 |
| Death vs survival* | 5/69 | 2/7 | 0.25 | (0.07, 1.07) | 0.12 |
| Lost to follow up vs all other outcomes^ | 6/69 | 0/7 | 0.01 | (-0.34,0.08) | 1.00 |
| **15-30** |  |  |  |  |  |
| Success vs all other outcomes | 664/996 | 87/133 | 1.01 | (0.88, 1.16) | 0.90 |
| Failure or recurrence versus al other outcomes* | 12/996 | 1/133 | 1.60 | (0.27, 9.59) | 1.00 |
| Death versus survival | 108/996 | 21/133 | 0.71 | (0.45, 1.13) | 0.15 |
| Lost to follow up versus all other outcomes | 212/996 | 24/133 | 1.20 | (0.8, 1.81) | 0.38 |
| **30-50** |  |  |  |  |  |
| Success vs all other outcomes | 1533/2373 | 298/455 | 0.97 | (0.91, 1.05) | 0.48 |
| Failure or recurrence versus al other outcomes | 25/2373 | 6/455 | 0.89 | (0.36, 2.17) | 0.80 |
| Death versus survival | 459/2373 | 94/455 | 0.97 | (0.79, 1.18) | 0.74 |
| Lost to follow up versus all other outcomes | 356/2373 | 57/455 | 1.20 | (0.92, 1.57) | 0.17 |
| **50-65** |  |  |  |  |  |
| Success vs all other outcomes | 395/654 | 123/175 | 0.85 | (0.76, 0.95) | 0.0054 |
| Failure or recurrence versus al other outcomes* | 8/654 | 2/175 | 1.07 | (0.26, 4.44) | 1.00 |
| Death versus survival | 199/654 | 39/175 | 1.39 | (1.03, 1.89) | 0.031 |
| Lost to follow up versus all other outcomes | 52/654 | 11/175 | 1.28 | (0.68, 2.24) | 0.44 |
| **Over 65** |  |  |  |  |  |
| Success vs all other outcomes | 56/152 | 26/47 | 0.69 | (0.49, 0.97) | 0.032 |
| Failure or recurrence versus al other outcomes^ | 1/152 | 0/47 | 0.01 | (-0.07,0.04) | 1.00 |
| Death versus survival | 82/152 | 20/47 | 1.23 | (0.85, 1.78) | 0.27 |
| Lost to follow up versus all other outcomes* | 13/152 | 1/47 | 4.02 | (0.72, 23.82) | 0.19 |

aRR=adjusted relative risk.

RR is presented as an adjusted figure following propensity score weighting, if less than five events occurred in one or both cohorts, outcome is presented as an unadjusted relative risk. If no events occurred in one or both cohorts the risk difference is presented.

*Denotes outcome presented as unadjusted relative risk

^Denotes outcome presented as risk difference

**Table S8: Primary analysis outcomes comparing patients treated using a short all-oral regimen without linezolid to patients treated with a short all-oral regimen including linezolid, as presented to the guideline development group for WHO guideline review process**

|  |  |  | **Risk of treatment outcome in the linezolid group compared with the ethionamide group** | | |
| --- | --- | --- | --- | --- | --- |
|  | **Linezolid group** | **All oral non linezolid group** | **aRR** | **95% CI** | **p value** |
| **Outcome** | **Events/Total** | **Events/Total** |  |  |  |
| **All Cases** |  |  |  |  |  |
| Success vs all other outcomes | 2705/4244 | 581/880 | 0.96 | (0.91, 1.01) | 0.11 |
| Failure or recurrence versus all other outcomes | 47/4244 | 12/880 | 0.81 | (0.42, 1.53) | 0.51 |
| Death vs survival | 853/4244 | 185/880 | 1.03 | (0.89, 1.20) | 0.66 |
| Lost to follow up vs all other outcomes | 639/4244 | 102/880 | 1.19 | (0.98, 1.45) | 0.084 |
| **PLHIV** |  |  |  |  |  |
| Success vs all other outcomes | 1759/2826 | 411/637 | 0.97 | (0.91, 1.04) | 0.40 |
| Failure or recurrence versus al other outcomes | 25/2826 | 9/637 | 0.6 | (0.28, 1.29) | 0.19 |
| Death versus survival | 629/2826 | 137/637 | 1.07 | (0.90,1.26) | 0.46 |
| Lost to follow up versus all other outcomes | 413/2826 | 80/637 | 1.08 | (0.86,1.35) | 0.52 |
| **HIV negative** |  |  |  |  |  |
| Success vs all other outcomes | 939/1406 | 169/242 | 0.92 | (0.84, 1.00) | 0.060 |
| Failure or recurrence versus al other outcomes* | 22/1406 | 3/242 | 1.26 | (0.41, 3.95) | 1.00 |
| Death versus survival | 221/1406 | 48/242 | 0.97 | (0.72,1.32) | 0.85 |
| Lost to follow up versus all other outcomes | 224/1406 | 22/242 | 1.62 | (1.05,2.51) | 0.029 |
| **AFB smear positive at baseline** |  |  |  |  |  |
| Success vs all other outcomes | 931/1451 | 221/341 | 0.97 | (0.89, 1.06) | 0.49 |
| Failure or recurrence versus al other outcomes | 23/1451 | 5/341 | 1.05 | (0.39, 2.82) | 0.92 |
| Death versus survival | 278/1451 | 81/341 | 0.89 | (0.71,1.12) | 0.31 |
| Lost to follow up versus all other outcomes | 219/1451 | 34/341 | 1.42 | (1.00, 2.01) | 0.048 |
| **AFB smear negative at baseline** |  |  |  |  |  |
| Success vs all other outcomes | 1608/2379 | 313/450 | 0.98 | (0.91, 1.05) | 0.54 |
| Failure or recurrence versus al other outcomes | 18/2379 | 6/450 | 0.54 | (0.21, 1.37) | 0.19 |
| Death versus survival | 392/2379 | 81/450 | 0.96 | (0.76,1.20) | 0.70 |
| Lost to follow up versus all other outcomes | 361/2379 | 50/450 | 1.24 | (0.94,1.65) | 0.13 |
| **Previous treatment for TB** |  |  |  |  |  |
| Success vs all other outcomes | 964/1654 | 229/371 | 0.95 | (0.86, 1.04) | 0.25 |
| Failure or recurrence versus al other outcomes | 25/1654 | 9/371 | 0.62 | (0.29, 1.33) | 0.22 |
| Death versus survival | 377/1654 | 90/371 | 0.97 | (0.79, 1.19) | 0.76 |
| Lost to follow up versus all other outcomes | 288/1654 | 43/371 | 1.40 | (1.04,1.9) | 0.028 |
| **No previous treatment for TB** |  |  |  |  |  |
| Success vs all other outcomes | 1741/2590 | 352/509 | 0.96 | (0.90, 1.03) | 0.25 |
| Failure or recurrence versus al other outcomes* | 22/2590 | 3/509 | 1.44 | (0.46, 4.51) | 0.79 |
| Death versus survival | 476/2590 | 95/509 | 1.10 | (0.89,1.35) | 0.38 |
| Lost to follow up versus all other outcomes | 351/2590 | 59/509 | 1.06 | (0.81,1.37) | 0.69 |
| **Isoniazid resistant#** |  |  |  |  |  |
| Success vs all other outcomes | 1117/1706 | 242/338 | 0.90 | (0.84, 0.97) | 0.010 |
| Failure or recurrence versus al other outcomes | 31/1706 | 7/338 | 0.94 | (0.41, 2.14) | 0.87 |
| Death versus survival | 296/1706 | 52/338 | 1.28 | (0.97, 1.7) | 0.08 |
| Lost to follow up versus all other outcomes | 262/1706 | 37/338 | 1.27 | (0.92, 1.77) | 0.15 |
| ***Isoniazid sensitive^+^*** |  |  |  |  |  |
| Success vs all other outcomes | 930/1421 | 217/346 | 1.04 | (0.95, 1.14) | 0.37 |
| Failure or recurrence versus al other outcomes* | 13/1421 | 3/346 | 1.06 | (0.33, 3.44) | 1.00 |
| Death versus survival | 269/1421 | 86/346 | 0.80 | (0.64, 0.99) | 0.041 |
| Lost to follow up versus all other outcomes | 209/1421 | 40/346 | 1.19 | (0.86, 1.64) | 0.29 |
|  |  |  |  |  |  |

aRR=adjusted relative risk. AFB=acid fast bacilli. PLHIV= people living with HIV.

RR is presented as an adjusted figure following propensity score weighting, if less than 5 events occurred in one or both cohorts, outcome is presented as an unadjusted relative risk. If no events occurred in one or both cohorts the risk difference is presented.

*Denotes outcome presented as unadjusted relative risk

^Denotes outcome presented as risk difference

#isoniazid resistance diagnosed on genotypic and phenotypic testing results

+isoniazid sensitivity only diagnosed on genotypic results; no phenotypic results available

Data presented to the guideline development group (GDG) for the review of WHO MDR-TB guidelines in 2022 is presented above. This cohort contained 63 patients in the comparison group who were excluded in the main analysis of this paper, as they did not receive ethionamide. These patients were included in the data presented to the GDG to answer the specific PICO question, “Should a shorter all-oral regimen (less than 12 months) containing at least three Group A medicines be used in patients with MDR/RR-TB and fluoroquinolone resistance excluded?”, therefore ethionamide was not a specific focus of this question. However, for this paper a comparison of ethionamide with linezolid in a short regimen was the predominant focus, therefore these patients were removed.

**
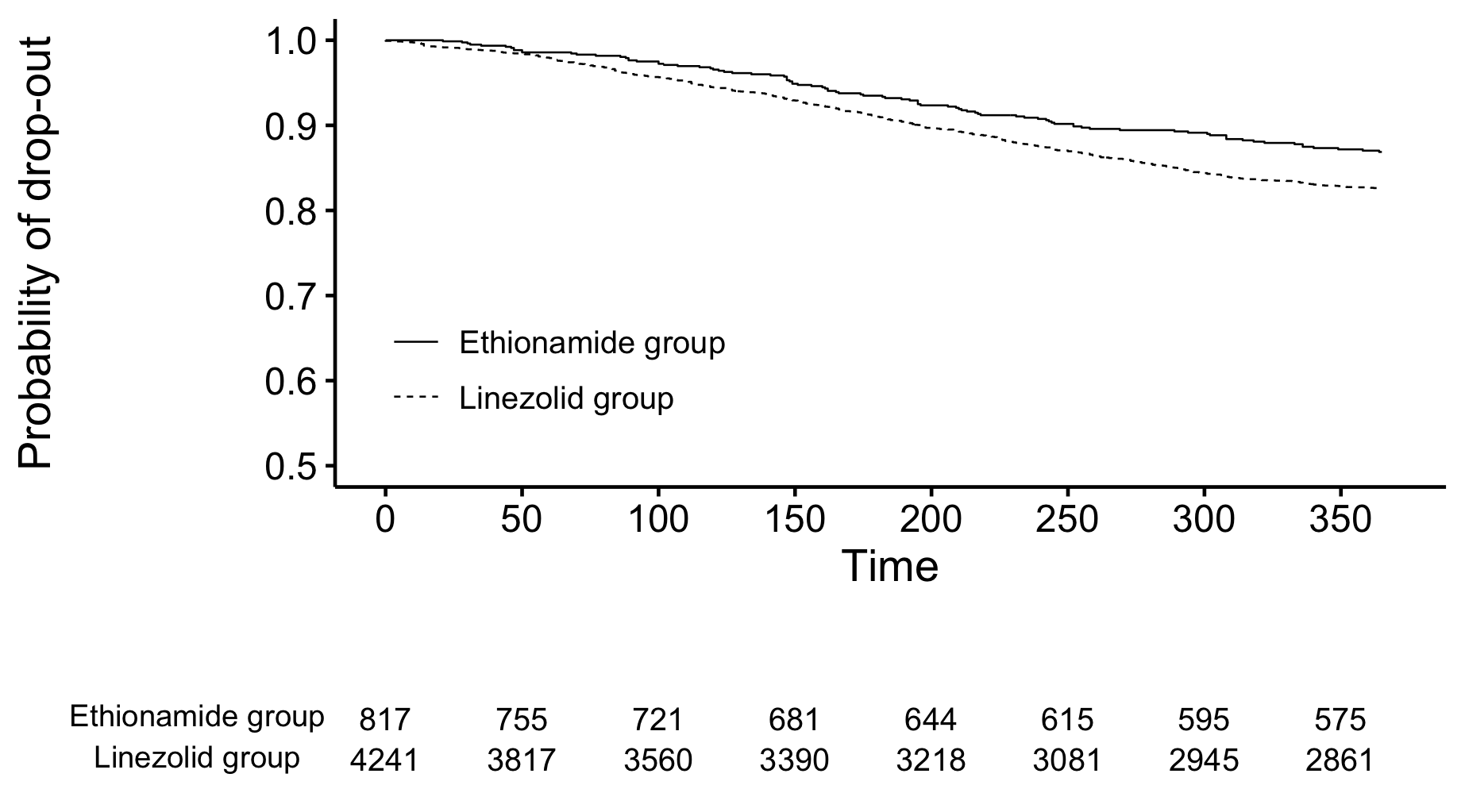
**

**Figure S1: Kaplan Meier survival estimate for time to loss to follow up by treatment group**

The results of this analysis are presented in the form of a Kaplan Meier curve (figure S1). Subjectively, the curves for each treatment group are very similar early in treatment. The proportion lost to follow up was the same in both groups at 50 days, with 2% lost to follow up in both groups, by 100 days this had increased to 3% in the ethionamide group and 4% in the linezolid group. This coincides with the time that late treatment toxicity is commonly observed with linezolid therapy, because of cumulative toxicity. This phenomenon is well recognised and justifies the approach taken in other trials to reduce the dose of linezolid after 16 weeks (6).

**References**

1 National Department of Health, South Africa. Interim clinical guidance for the implementation of injectable-free regimens for rifampicin-resistant tuberculosis in adults, adolescents and children. 2018. <https://www.tbonline.info/media/uploads/documents/dr_tb_clinical_guidelines_for_rsa_september_2018.pdf> (accessed 6 Jan 2023)

2 Chesnaye NC, Stel VS, Tripepi G, et al. An introduction to inverse probability of treatment weighting in observational research. *Clinical Kidney Journal* 2021; **15**: 14­–20.

3 White IR, Royston P, Wood AM. Multiple imputation using chained equations: Issues and guidance for practice. *Statistics in medicine* 2011; **30**: 377–99.

4 Van Buuren S, Groothuis-Oudshoorn K (2011). mice: Multivariate Imputation by Chained Equations in R. *Journal of Statistical Software* 2011; **45**: 1–67.

5 Granger E, Sergeant J, Lunt M. Avoiding pitfalls when combining multiple imputation and propensity scores. *Statistics in Medicine* 2019; **38**: 5120–32.

6 Nyang’wa BT, Kazounis C, Motta E, et al. (2022). A 24-Week, All-Oral Regimen for Rifampin-Resistant Tuberculosis. *New England Journal of Medicine* 2022; **387**: 2331-2343.
